# Detection of Cardiac Biomarkers Using Single Polyaniline Nanowire-Based Conductometric Biosensors 

**DOI:** 10.3390/bios2020205

**Published:** 2012-05-14

**Authors:** Innam Lee, Xiliang Luo, Jiyong Huang, Xinyan Tracy Cui, Minhee Yun

**Affiliations:** 1 Department of Electrical and Computer Engineering, University of Pittsburgh, Pittsburgh, PA 15261, USA; E-Mails: inl8@pitt.edu (I.L.); jih44@pitt.edu (J.H.); 2 Department of Bioengineering, University of Pittsburgh, Pittsburgh, PA 15260, USA; E-Mails: xiliangluo@hotmail.com (X.L.); xic11@pitt.edu (X.T.C.)

**Keywords:** myoglobin, cardiac troponin I, creatine kinase-MB, b-type natriuretic peptide, polyaniline, nanowire, conductometric biosensing

## Abstract

The detection of myoglobin (Myo), cardiac troponin I (cTnI), creatine kinase-MB (CK-MB), and b-type natriuretic peptide (BNP) plays a vital role in diagnosing cardiovascular diseases. Here we present single site-specific polyaniline (PANI) nanowire biosensors that can detect cardiac biomarkers such as Myo, cTnI, CK-MB, and BNP with ultra-high sensitivity and good specificity. Using single PANI nanowire-based biosensors integrated with microfluidic channels, very low concentrations of Myo (100 pg/mL), cTnI (250 fg/mL), CK-MB (150 fg/mL), and BNP (50 fg/mL) were detected. The single PANI nanowire-based biosensors displayed linear sensing profiles for concentrations ranging from hundreds (fg/mL) to tens (ng/mL). In addition, devices showed a fast (few minutes) response satisfying respective reference conditions for Myo, cTnI, CK-MB, and BNP diagnosis of heart failure and for determining the stage of the disease. This single PANI nanowire-based biosensor demonstrated superior biosensing reliability with the feasibility of label free detection and improved processing cost efficiency due to good biocompatibility of PANI to monoclonal antibodies (mAbs). Therefore, this development of single PANI nanowire-based biosensors can be applied to other biosensors for cancer or other diseases.

## 1. Introduction

The incidence of myocardial infarction, which has one of the highest mortality rates in the US and Europe, increases in elderly people [1,2]. Therefore, the diagnosis and prevention of all cardiac disorders is very important. For the detection of myocardial infarction, myoglobin (Myo), cardiac troponin I (cTnI), creatine kinase-MB (CK-MB), and b-type natriuretic peptide (BNP) have been selected as biomarkers for the diagnosis [1,3,4]. Among those cardiac markers, Myo is the fundamental protein to check at the onset of infarction [1,5]. However, it has cross-activity with skeletal muscle pain [6]. Therefore, it is necessary to monitor the level of other proteins such as cTnI, CK-MB, and BNP in patients’ serum for accurate, prompt and continuous diagnosis of myocardial infarction [2,3,4]. cTnI is only specific to cardiac muscles and never found in healthy people [7]. CK-MB and BNP are related to recurrence of myocardial infarction and cardiac vascular disease, respectively [1,7]. 

The detection of cardiac biomarkers has been investigated using several methods such as fluorescence [8,9], surface plasma resonance (SPR) [10,11], and electrical signals from nanowire-based biosensors [12,13]. For examples, biosensing based on fluorescence has been applied for the detection of Myo, which was carried out to measure fluorescent intensity from sandwich immunoassay labeled with fluorescent dyes [14]. In addition, SPR, which measures SPR angle shift once target proteins are bound on specifically functionalized substrates, is one of the most popular biosensing methods to be employed for various cardiac markers such as Myo, cTnI, and BNP [5,11,15]. Although the previously developed biosensors utilizing fluorescence or SPR have shown effective performances, these methods have some limitations in sensitivity, miniaturization and cost efficiency. They have relatively lower sensitivity and specificity than nanomaterial-based biosensors such as nanoparticles, carbon nanotubes (CNTs), and nanowires [16,17,18]. Those nanomaterials provide outstanding physical properties such as tunable conductivity by doping and synthesis methods, and high carrier mobility to realize real-time sensing in 0- or 1-dimensional structure [19,20]. To date, these advantages of nanomaterials have been actively studied to develop biosensors based on inorganic or organic nanomaterials. Inorganic nanomaterials such as Si nanowires and CNT have been fabricated through various methods and developed for the applications of electrical devices, chemical sensors, and biosensors [21,22,23]. For example, Si nanowire sensor arrays were developed to detect very low concentrations of cTnI by monitoring the change of conductance on the nanowire biosensor [13].

Biosensors based on inorganic nanomaterials require complicated processing conditions for functionalization with bio-recognition elements such as antibodies due to the low-biocompatibility of inorganic nanomaterials. In contrast, organic nanomaterials such as polyaniline (PANI) and polypyrrole (PPy) are more easily modified with biomolecules than inorganic nanomaterials [24,25,26]. During the functionalization of the PANI surface, the covalent bond between PANI and the antibody enables the direct measurement of the physical change of conductance, capacitance, or impedance upon the binding of antibodies to target proteins [27,28]. In addition, conducting polymers such as PANI or PPy are appealing for electrical, mechanical, or biomedical applications due to the advantages of controllable conductivity, mechanical flexibility, and exceptional bioaffinity [29]. Furthermore, the PANI or PPy nanowires have been applied in the organic nanowire field effect transistor (FET), light emission diode, and biosensor [30,31,32,33,34]. However, most of these applications were developed based on bundled nanowires and required selection and alignment procedures that are time-consuming and lower the production yields. 

In this research, we report the development of single PANI nanowire-based biosensors for detecting four cardiac biomarkers: Myo, cTnI, CK-MB, or BNP. The single PANI nanowire was directly fabricated via the electrochemical deposition growth method between pre-patterned Au electrodes, avoiding the need for the selection and alignment of the nanowire [35,36]. For the functionalization of the fabricated single PANI nanowires, the mAbs of the aforementioned cardiac markers were covalently attached to PANI nanowires by a surface immobilization method. After the PANI functionalization, the biosensing of cardiac biomarkers was carried out by measuring the conductance change of the nanowires. The conductance of the PANI nanowire was monitored in the various conditions of the functionalized PANI nanowire, after injection of phosphate buffer saline (PBS), bovine serum albumin (BSA), and target biomarkers. The conductance of the nanowire can be modulated by the major carrier accumulation or depletion. The binding between immobilized mAbs and target biomarkers changes the net surface charge of the single PANI nanowire and induces the carrier accumulation or depletion depending on the values of net surface charge and types of nanowire. In addition, the nanowire shows no conductance change to BSA or non-target proteins due to the mAbs specificity. 

In order to study the biosensing performance, the single PANI nanowire-based biosensor was tested in the broad range from tens (fg/mL) to (ng/mL) of cTnI, CK-MB, and BNP proteins and showed linear sensitivity along different concentrations with a small standard deviation of less than 15%. In addition, the detection of cardiac biomarkers also showed a remarkable specificity value of over 10^6^ fold, where specificity is defined as the ratio of (the highest concentration of non-specific protein showing ignorable or non-response signal) to (the lowest concentration of specific protein showing significant signal change) in the test of BSA or other cardiac markers. The measurement of conductance facilitates fast response in a few minutes, while a conventional method like immunoassay requires at least a few hours to incubate the complex of mAbs and targets [37]. Furthermore, integration of microfluidic channels on the nanowire biosensors allows more accurate sensing and slow flow of sample solution only through the active area of the PANI nanowire [38].

## 2. Experimental Section

### 2.1. Materials

Ionic aniline solution (0.01 M aniline in 0.1 M HCl) was prepared for nanowire fabrication. All human cardiac biomarkers (Myo, cTnI, CK-MB, and BNP) and the corresponding mAbs (Myo-mAbs, cTnI-mAbs, CK-mAbs, and BNP-mAbs) were purchased from Abcam and Sigma-Aldrich. The BNP used in this research has 32 amino acids. For the surface immobilization of mAbs on the fabricated single PANI nanowires, ethyl(dimethylaminopropyl) carbodiimide (EDC, 0.2 M) and N-Hydroxysuccinimide (NHS, 0.2 M), and BSA (1 ng/mL–2 mg/mL) of certified analytical grade were purchased from Sigma-Aldrich and used without further purification. PBS (10 mM phosphate, pH 7.4) was introduced as washing and working buffer solution.

### 2.2. Fabrication of Single PANI Nanowire

A 5 µm long single PANI nanowire was fabricated in a nanochannel bridging two metal electrodes through electrochemical deposition. First, Au electrodes were patterned lithographically and deposited using an e-beam evaporator (VE-180, Thermionics) on a Si/SiO_2_ substrate. The nanochannel with a width of 100 nm and depth of 100 nm was made between a pair of electrodes on the layer of polymethyl methacrylate (PMMA), which is coated using e-beam lithography (e-line, Raith). After preparing the nanochannel, a static current of 500 nA was applied to induce the electrochemical deposition of PANI along the nanochannel from the ionic aniline solution. The change of voltage between the two metal electrodes was monitored by a semiconductor analyzer (B1500A, Agilent). The drop of voltage to sub-micro voltage indicates completion of the fabrication of the single PANI nanowire. The substrate including the single PANI nanowires were soaked in acetone to remove the PMMA layer for 10 min. This electrochemical deposition growth method is explained in detail elsewhere [36]. 

### 2.3. Functionalization of Single PANI Nanowire

The fabricated single PANI nanowire was functionalized for a cardiac biosensor in order to detect cardiac biomarkers through the surface immobilization method utilizing EDC/NHS solution. The EDC/NHS solution with mAbs of target biomarkers assists to form covalent bonds between PANI and mAbs. The mixture of EDC/NHS and mAbs were prepared at three different concentrations (50, 100, and 200 µg/mL) for the optimization of functionalization in order to obtain the highest sensitivity of biosensor and linear sensing profile. Before functionalization, the single PANI nanowires were soaked in 0.1 M HCl for 10 min, then in the mixture solution of EDC/NHS and mAbs for 3 h at room temperature. After washing the functionalized PANI nanowires with PBS and de-ionized water (18.2 MΩ) to remove un-immobilized mAbs, the nanowires were immersed in 2 mg/mL BSA for blocking the non-reacted functional groups for 30 min. This was followed by another washing with PBS and de-ionized water to clean un-coated BSA on the surface of the nanowires. Finally, those single PANI nanowires were utilized for the cardiac biosensors.

### 2.4. Preparation of Microfluidic Channel

**Figure 1:**
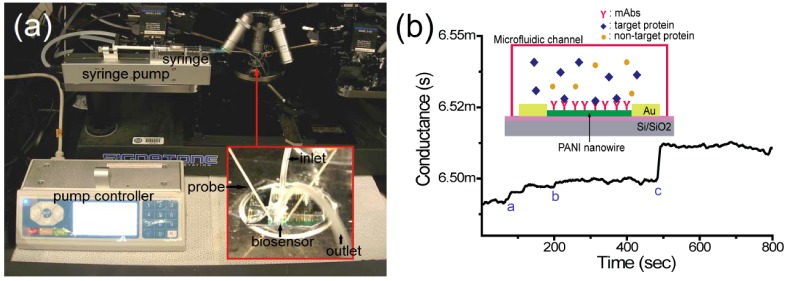
An illustration and the experimental set-up of the single polyaniline (PANI) nanowire biosensor to detect cardiac biomarkers. (**a**) The experimental setup; the microfluidic channel is adhered on the nanowire biosensor and the nanowire biosensor chip is mounted on a probe station connected to the semiconductor analyzer and syringe pump with inlet and outlet; (**b**) The conductance change in the single PANI nanowire-based biosensor is monitored. The injection of PBS (mark a), BSA (mark b), and cardiac biomarker (mark c) shows the different changes of conductance.

A microfluidic channel was fabricated using polydimethylsiloxane (PDMS, Sylgard 184, Dow Corning Corp.) and negative photoresist (SU-8 2050, MicroChem Corp.). A designed mold of the microfluidic channel was lithographically patterned and developed on a Si/SiO_2_ wafer with spin-coated SU-8 2050 of 100 µm thickness. The fabricated microfluidic channels are 700 µm in width, 100 µm in height, and 4 mm in length and these dimensions are determined by the diameters of fluidic tube and syringe needle. The prepared PDMS was poured on the mold of the microfluidic channel and cured in an oven at 80 °C for 45 min. The fabricated PDMS microfluidic channel was adhered to a nanowire biosensor chip after O_2_ plasma treatment (250 mT, 30 W, 30 s) as shown in the inset of Figure 1(a). The single PANI nanowire biosensor integrated with the microfluidic channel was tested by infusing PBS, BSA, or target solutions using a syringe pump with the flow rate of 0.03 mL/min.

### 2.5. Detection of Target Proteins on the Nanowire Biosensor

The detection of cardiac biomarkers was carried out using the conductometric sensing method by measuring the conductance change of the nanowire. After adhesion of the microfluidic channel on the functionalized PANI nanowires, the nanowire biosensor was connected to the semiconductor analyzer through a probe station as shown in Figure 1(a).

In order to inject PBS or target protein solutions, a micro-tube was connected from an inlet of the microfluidic channel to the syringe pump. Another micro-tube was connected with the other end of the microfluidic channel as an outlet to withdraw PBS or target protein solutions as shown in the inset of Figure 1(a). The change of conductance on the nanowire was measured by applying a static current of 50 µA and sampling ratio of 2 Hz with the semiconductor analyzer. Using a flow rate of 0.03 mL/min, laminar flow was established in the micro-tube and microfluidic channel, preventing nanowire breakage and conductance variation due to turbulence. In conductometric biosensing on the single PANI nanowire, first, a baseline of conductance was obtained from the flowing PBS solution (mark a) as shown in Figure 1(b). Once the conductance was stabilized in 300 s after injection of PBS solution into the microfluidic channel, the high concentration of BSA (mark b) was applied for the test of specificity in the nanowire biosensor. When the solution reaches the PANI nanowire and fills out the microfluidic channel, the conductance of the nanowire shows little change from the baseline of conductance. However, the injection of the target biomarker (mark c) shows a clear change of conductance value due to the binding of mAbs with target biomarkers as depicted in the inset of Figure 1(b).

## 3. Results and Discussion

### 3.1. Functionalization of Single PANI Nanowires with mAbs

**Figure 2:**
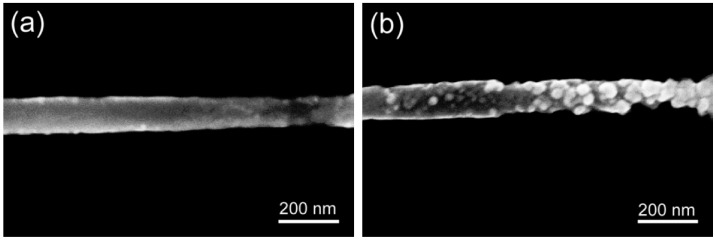
Scanning electron microscopy (SEM) images of single PANI nanowires. (**a**) before the surface functionalization and (**b**) after the surface functionalization with cTnI mAbs. The two SEM images were taken at the same location of the nanowire.

The same single PANI nanowires were compared by scanning electron microscopy (SEM) before and after the surface immobilization of mAbs to observe the change of nanowire surfaces as shown in Figure 2(a,b). In the SEM images, the observed difference of PANI nanowire surface distinguishes the functionalized nanowire from the non-functionalized nanowire. Before the functionalization, the single nanowire has a smooth surface and uniform dimension with a width of 100 nm as shown in Figure 2(a). In contrast, the surface of the functionalized single PANI nanowire shows a rough morphology with attached particles of 10–30 nm in diameter in Figure 2(b).

During the functionalization of the nanowire, several washing processes with PBS and de-ionized water eliminate un-immobilized mAbs from the surface of the nanowire and the substrate including the Au electrodes and SiO_2_ layer. Based on these observations in Figure 2, the size of these particles is consistent with the average size of antibodies [39,40]. Therefore, we conjecture that the immobilization of mAbs with EDC/NHS solution allows strong binding between the PANI nanowire and mAbs due to this difference on the surface of the nanowire after the washing process, discussed also in other researches [41,42]. For the further verification of the surface immobilization, various methods such as the characterization of chemical bond changes and the observation of labeled immobilized antibodies with fluorescent materials or nanoparticles has been employed [43,44]. In our experiments, the surface immobilization methods of mAbs have been verified with fluorophore-tagged immunoglobulin G (IgG) mAbs and Raman spectroscopy [45,46]. The immobilized fluorophore-tagged IgG mAbs emitted red fluorescent light on the only nanowire excluding Au electrodes or the SiO_2_ layer. In addition, the Raman spectroscopy showed the presence of 1,638 cm^−1^ and 1,240 cm^−1^ bands from Amide groups, providing the immobilization of the IgG mAbs on the PANI nanowire. The functionalization only occurs on the single PANI nanowire not the Au electrodes and SiO_2_ layer of the biosensor chip. This approach eliminates the need for any passivation layer, which was required to prohibit signal interference from electrodes or substrate in studies using inorganic nanomaterials [23,38]. The surface immobilization method using EDC/NHS provides an efficient functionalization process of the single PANI nanowire, reducing process steps and the passivation layer unlike inorganic materials-based biosensors.

### 3.2. Detection of Cardiac Biomarkers

**Figure 3:**
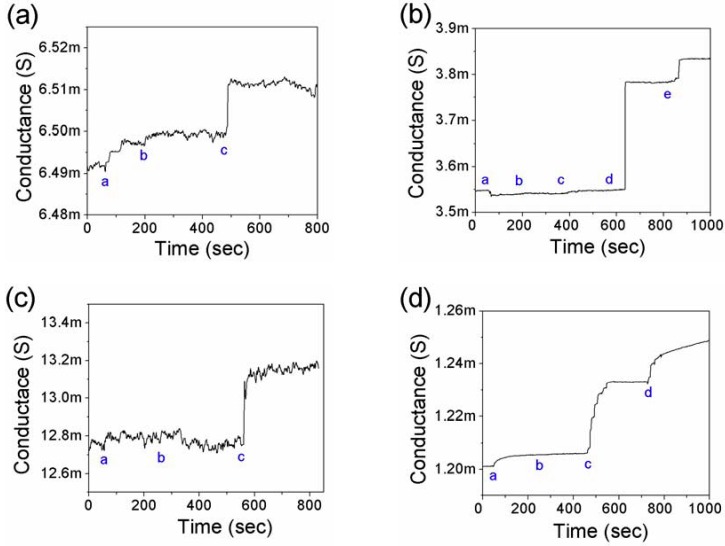
Single PANI nanowire biosensor chips integrated with microfluidic channel present the lowest detection of cardiac biomarkers. (**a**) Detection of Myo (a: PBS, b: 100 ng/mL BSA, and c: 100 pg/mL Myo); (**b**) Detection of cTnI (a: PBS, b: 10 ng/mL BSA, c: 5 fg/mL cTnI, d: 250 fg/mL cTnI, e: 20 pg/mL cTnI); (**c**) Detection of CK-MB (a: PBS, b: 10 ng/mL BSA, c: 150 fg/mL CK-MB); (**d**) Detection of BNP (a: PBS, b: 100 ng/mL BSA, c: 50 fg/mL BNP, and d: 1 pg/mL BNP).

The detection of Myo, cTnI, CK-MB, and BNP on the single PANI nanowire biosensors was carried out by monitoring the change of conductance in the nanowires as shown in Figure 3. The integration of the microfluidic channel assists accurate and reliable biosensing by directing the flow of the solution only onto the active area of the single PANI nanowire and minimizing the damage of the nanowire with a slow flow rate. In addition, the lowest detections of Myo, cTnI, CK-MB, and BNP could be obtained at 100 pg/mL, 250 fg/mL, 150 fg/mL, and 50 fg/mL as demonstrated in Figure 3(a–d), respectively. 

**Figure 4:**
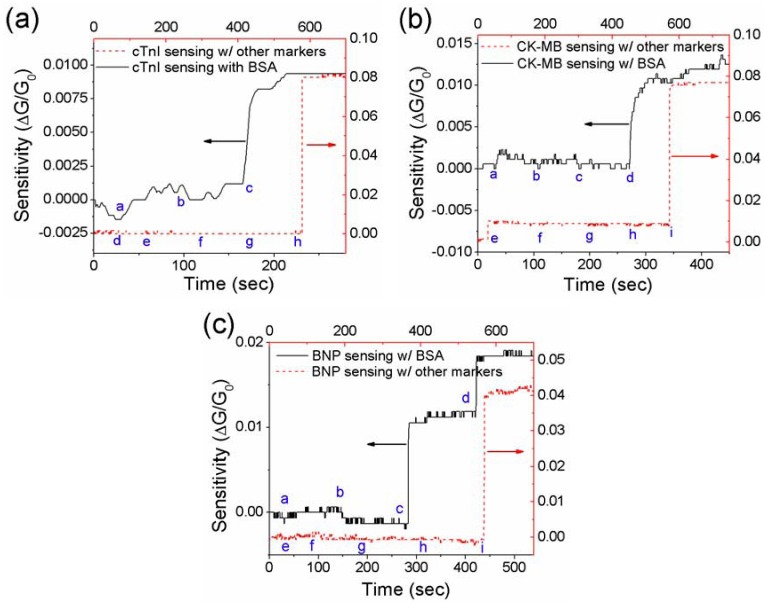
Specificity tests of the single PANI nanowire biosensor in the presence of non-target proteins. (**a**) For detection of cTnI (a: PBS, b: 1 ng/mL BSA, c: 500 fg/mL cTnI, d: PBS, e: 1 ng/mL Myo, f: 1 ng/mL CK-MB, g: 1 ng/mL BNP, and h: 1 ng/mL cTnI), the nanowire biosensor responds to only cTnI; (**b**) For detection of CK-MB (a: PBS, b: 1 ng/mL BSA, c: 100 ng/mL BSA, d: 25 pg/mL CK-MB, e: PBS, f: 1 ng/mL Myo, g: 1 ng/mL cTnI, h: 1 ng/mL BNP, and i: 1 ng/mL CK-MB), the nanowire biosensor responds to only CK-MB; (**c**) For detection of BNP (a: PBS, b: 100 ng/mL BSA, c: 1 ng/mL BNP, d: 10 ng/mL BNP, e: PBS, f: 1 ng/mL Myo, g: 1 ng/mL cTnI, h: 1 ng/mL CK-MB, and i: 1 ng/mL BNP), the nanowire biosensor responds to only BNP.

This detection limit of Myo supported with the microfluidic channel is much lower than our previous result of 1.3 ng/mL and shows ultra-high specificity to BSA of 100 ng/mL [45]. In these tests, the specificity values were calculated in the range from 1 × 10^4^ fold in Myo detection to 2 × 10^6^ fold in BNP detection (cTnI: 4 × 10^5^ fold and CK-MB: 6.7 × 10^5^ fold). These detection limits of cardiac biomarkers were measured in the absence of non-specific proteins; the biosensing of cardiac biomarkers was measured after the flow of non-specific protein solution into the microfluidic channels. In order to apply the biosensor for practical diagnosis, it is necessary to verify sensing performance in the presence of BSA or non-target cardiac biomarkers as shown in Figure 4.

The presence of non-target proteins may interfere with the sensing performance due to the screening or physical absorption of non-target proteins. The detection of cardiac biomarkers with BSA may provide similar conditions to the practical diagnosis, because serum albumin is one of the most abundant proteins in human serum. On the other hand, the biosensing with other cardiac biomarkers shows functionality to detect only specific target proteins depending on the immobilized mAbs. The biosensing of cardiac biomarkers for cTnI, CK-MB, and BNP with non-target proteins are demonstrated respectively in Figure 4(a–c). Each nanowire biosensor was tested with BSA at a concentration of 1–100 ng/mL followed by each target protein, showing a significant conductance change as shown in Figure 4 (black solid line). The nanowire biosensors were tested with the addition of other cardiac biomarkers (red dash line) and show good specificity to detect only the target biomarkers. In the presence of non-target proteins, the nanowire biosensors have around 1 × 10^3^–1 × 10^6^ fold specificity values, which are lower than the specificity values in the test with the integrated microfluidic channel but acceptable for biosensing applications. Non-specific binding of non-target proteins is restrained by the blocking process with BSA (2 mg/mL) on the surface of the nanowire after the functionalization process. The concentration of BSA blocking solution was considered to cover only the area unoccupied by mAbs without losing biosensing activity [47]. Therefore, a satisfying level of specificity was obtained and the developed single PANI nanowire-based biosensors demonstrated to be feasible to detect cardiac biomarkers under conditions where the target biomolecules are together with a high concentration of non-target biomolecules.

**Figure 5:**
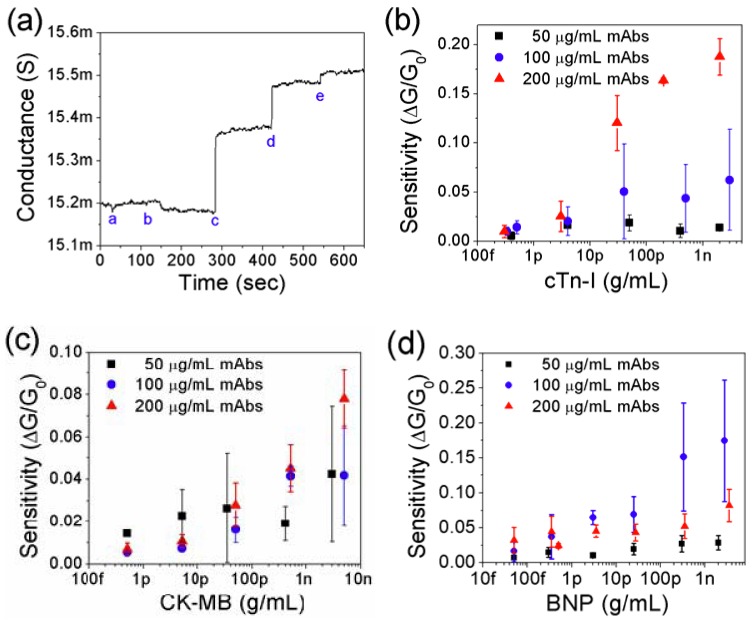
Biosensing of cardiac biomarkers in a broad sensing range and optimization of sensing performance. (**a**) Stepwise change of conductance according to introducing different concentrations of BNP to the nanowire biosensor (a: PBS, b: 100 ng/mL BSA, c: 1 ng/mL BNP, d: 10 ng/mL BNP, and e: 100 ng/mL BNP); (**b**) In order to optimize the condition of functionalization, sensitivities of the nanowire biosensors are compared in different concentrations of cTnI mAbs. 200 µg/mL cTnI mAbs presents the best linear sensing profile and the highest sensitivity of the three different conditions of cTnI mAbs; (**c**) For CK-MB, 200 µg/mL CK-MB mAbs shows the best sensing profile without fluctuation of sensitivity; (**d**) For BNP, 100 µg/mL BNP mAbs provide higher sensitivity in the broad sensing range than other conditions.

In the biosensing of cardiac biomarkers, it is crucial that a biosensor has a broad range of detection for the diagnosis of heart disease. In order to investigate the sensing performance, various concentrations of BNP from 1 ng/mL to 100 ng/mL were introduced to the single PANI nanowire biosensor as shown in Figure 5(a). Above the baseline of conductance in PBS (mark a), the nanowire biosensor shows noticeable conductance changes along the different concentration of BNP as demonstrated in Figure 5(a); (b): 100 ng/mL BSA; (c): 1 ng/mL BNP; (d): 10 ng/mL BNP, and (e): 100 ng/mL BNP. The increased concentration of BNP provides a stronger charge effect due to accumulation of holes in the PANI nanowire. However, the continuous biosensing tests with several different concentrations of cardiac biomarkers consume the detectable mAbs and make the change of conductance become small with the saturation of conductance. During the biosensing of BNP, the change of conductance occurs within a few minutes after the introduction of the target proteins solutions to the single PANI nanowire. In addition, the continuous biosensing tests for Myo, cTnI and CK-MB show similar results to BNP with increasing the concentration of cardiac biomarkers [45]. Therefore, our biosensing results for cardiac biomarkers indicate that the developed single PANI nanowire biosensors show a wide sensing range, required reference values, and fast response time required to provide label free emergency detection and diagnosis.

The sensing performance of the nanowire biosensor such as cost efficiency, sensitivity, and sensing reproducibility may be maximized by finding the optimum conditions of functionalization (concentrations of 50, 100, and 200 µg/mL for each mAbs) as shown in Figure 5(b–d). The biosensing tests were carried out at least 3 times using different nanowires at each concentration to avoid the issue of sensitivity loss due to multiple biosensing tests in the same nanowire. In order to find optimal conditions with antibody concentration, we investigated various conditions of surface immobilization satisfying sensing performance and to realize cost-efficiency in the development of the nanowire biosensor. In cTnI mAbs of 50 and 100 µg/mL, the sensitivities of the nanowire biosensors remained at around the level of 0.02 and 0.05 over cTnI of 30 pg/mL as shown in Figure 5(b). For cTnI sensing, functionalization using mAbs of 200 µg/mL showed the highest sensitivity and broadest sensing range from 300 fg/mL to 3 ng/mL. Standard deviations under the condition of 200 µg/mL are much smaller than other conditions indicating the best reproducibility. Similarly to the test of cTnI, the optimizations of surface immobilization for CK-MB mAbs and BNP mAbs were carried out as shown in Figure 5(c,d), respectively. CK-MB of 200 µg/mL shows the relatively smaller deviation and more linearly increased sensitivity than other concentrations of CK-MB mAbs. BNP of 100 µg/mL shows linearly increased sensitivity along the broad range of BNP concentrations from 50 fg/mL to 3 ng/mL. However, the deviation in that condition is greater than for BNP mAbs (50 µg/mL and 200 µg/mL) as shown in Figure 5(d). Based on these tests, the optimal functionalization of single PANI nanowires is determined by the linear sensitivity in the broad range of target concentration and good sensing reproducibility with a small standard deviation of sensitivity. The various results from the optimization of functionalization may be caused by the size of mAbs, uniformity of the immobilized mAbs per unit area and orientation of the immobilized mAbs [48]. Considering the concentration of mAbs, if an insufficient amount of mAbs on the PANI nanowire were provided, an insufficient conduction change could result from the small net surface charge. On the other hand, if a high concentration of mAbs was employed, the plentiful active binding sites on the surface of the nanowire could improve sensing linearity and sensitivity. However, excessively immobilized mAbs in the functionalization of the nanowire may crosslink together between primary amines and carboxylic groups of the mAbs. This reaction results in less active binding sites and low sensitivity [49].

In Figure 5(b–d), the low concentrations (50 µg/mL, marked as solid black square) of each mAbs show the competitive sensitivity in the range from 50 fg/mL scale to 5 pg/mL scale. However, the sensitivities of the nanowire biosensors with mAbs of 50 µg/mL are very poor and show the saturation behavior in high concentrations of the target biomarkers. In these biosensing regions, the small number of binding sites from the immobilized mAbs result in the weak net surface charge to the single PANI nanowires for the detection in the high concentration of target biomarkers. The high concentrations of mAbs (100 or 200 µg/mL) have shown a relatively higher sensitivity and linear sensing profile than the mAbs of 50 µg/mL in this research. Therefore, plentiful binding sites on the functionalized nanowire is an important condition for realizing a high performance biosensor. However, the advantages of the single PANI nanowires-based biosensor will not include cost efficiency of the surface immobilization, if concentrations greater than 200 µg/mL mAbs are employed.

### 3.3. Effect of Net Surface Charge on the Single PANI Nanowire Biosensor

The single PANI nanowire biosensor for the detection of cardiac biomarkers has demonstrated high sensitivity, fast detection, and good sensing reproducibility. The use of conductometric measurement has the advantages of not requiring a reference electrode and low operating voltage [50,51]. During biosensing, the increased conductance is mainly caused by charge carrier accumulation on the P-type PANI nanowire through binding of the charged target proteins to the immobilized mAbs on the surface of the PANI nanowire. 

**Figure 6:**
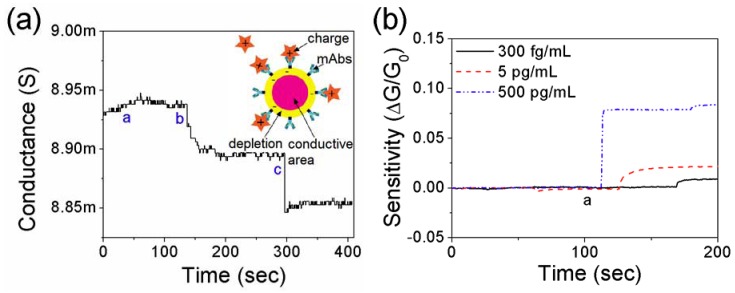
Tests of net surface charge effect on the functionalized PANI nanowires. (**a**) Decrease of conductance on the nanowire biosensor in sensing test with positively charged cTnI protein solutions (a: PBS of pH 5, b: 1 ng/mL, and c: 10 ng/mL). cTnI protein solutions were prepared with PBS of pH 5; (**b**) Comparison of sensitivity with different concentrations of cTnI detection. The nanowire biosensor shows significantly higher sensitivity with higher concentration. The mark “a” on black solid line presents the injection of BSA (100 ng/mL). After the injection of BSA, 300 fg/mL cTnI was injected into the biosensor.

The charge of the target protein solutions is related to the pH value of PBS, which is used as a buffer solution for the target protein, and the isoelectric point (pI) values of the proteins. It is generally known that Myo, cTnI, CK-MB, and BNP have pIs of 7.2, 5.2–5.4, 5.2, and 6.5 respectively [52,53,54]. The net charges of these target protein solutions in PBS (pH 7.4) are negative due to the pI values lower than pH 7.4. Based on our biosensing experiments and pI values of target proteins, it is assumed that the negative charges of target proteins resulted in a carrier accumulation on the PANI nanowire and consequently an increase of conductance. To verify this hypothesis, another cTnI solution in PBS of pH 5 was prepared and tested as shown in Figure 6(a). cTnI in PBS with pH 5 has positive charges due to a pI value of 5.2–5.4 and the binding to immobilized cTnI mAbs leads to carrier depletion in the PANI nanowire. Figure 6(a) shows that the conductance of the PANI nanowire decreased upon the addition of the cTnI solution. The inset of Figure 6(a) depicts the change of conductive area in the nanowire by depletion after binding the positive charged target protein to the mAbs.

The conductometric biosensing on the single PANI nanowire easily differentiates signal changes from very low concentrations to high concentrations of target proteins, determined by the electric field strength from the net charge in complexes of mAbs and target proteins. The tiny dimension of nanowire can be easily affected by the single molecular charge on the surface [55,56]. The complexes of mAbs and target cardiac biomarkers lead to charge neutralization and redistribution at the interface between the mAbs and the target proteins [57,58]. The opposite charges to the target proteins assemble at the top of mAbs while the same charges to the target proteins redistribute to the bottom of mAbs on the nanowire surface. The driven charges in the complexes of mAbs and proteins affect the accumulation or depletion of major carriers in the nanowire. In addition, the higher concentrations of charged proteins lead to higher sensitivity due to stronger potential from the complex of mAbs and proteins as compared in Figure 6(b). In these biosensing tests, the different concentrations of cTnI are compared in their sensitivity from different single PANI nanowires-based biosensors. Non-response to 100 ng/mL BSA (mark “a” on the black line in Figure 6(b)) demonstrates that non-specific proteins do not construct complexes with mAbs or pre-coated BSA on the free-site of the nanowire. Therefore, it is conjectured that the charge neutralization in complexes of mAbs and target proteins realizes conductometric biosensing and as low as 100 pg/mL Myo, 250 fg/mL cTnI, 150 fg/mL CK-MB, and 50 fg/mL BNP for detection limits. However, this conjecture includes partial shortcomings, and is insufficient to support high specificity in biosensing. Those aforementioned sensing mechanism and the specificity of the nanowire biosensor leave room for further investigation and discussion.

## 4. Conclusions

The detection of cardiac biomarkers was successfully carried out through use of the single PANI nanowire biosensor, showing ultra-high sensitivity, good sensing reproducibility and high specificity. The high specificity of above 10^6^ fold to BSA or other non-specific proteins showed the promising potential of using the single PANI nanowire biosensor for biomedical diagnosis. The integration of a microfluidic channel on the nanowire biosensor allows accurate detection of target proteins and very low detection limits of Myo, cTnI, CK-MB, and BNP, minimizing breakage of the nanowire, safe sample handling, and limiting the flow of target solutions only onto the nanowire. In addition, this microfluidic channel provides the advantages of sensing reliability and system stability due to the flow rate control in the laminar flow region. The design of the single PANI nanowire biosensor reported here can be applied for the detection of various other biomarkers such as cancer markers promising satisfaction in required sensing performance via the surface immobilization of mAbs using EDC/NHS.
